# Analysis of Microarray Data on Gene Expression and Methylation to Identify Long Non-coding RNAs in Non-small Cell Lung Cancer

**DOI:** 10.1038/srep37233

**Published:** 2016-11-16

**Authors:** Nannan Feng, Travers Ching, Yu Wang, Ben Liu, Hongyan Lin, Oumin Shi, Xiaohong Zhang, Min Zheng, Xin Zheng, Ming Gao, Zhi-jie Zheng, Herbert Yu, Lana Garmire, Biyun Qian

**Affiliations:** 1 Hongqiao International Institute of Medicine, Shanghai Tongren Hospital and Faculty of Public Health, Shanghai Jiao Tong University School of Medicine, Shanghai 200025, China; 2 Cancer Epidemiology Program, University of Hawaii Cancer Center, 701 Ilalo Street, Honolulu, HI 96813, USA; 3 Key Laboratory of Cancer Prevention and Therapy, Tianjin Medical University Cancer Institute and Hospital, Tianjin, 300060, China

## Abstract

To identify what long non-coding RNAs (lncRNAs) are involved in non-small cell lung cancer (NSCLC), we analyzed microarray data on gene expression and methylation. Gene expression chip and HumanMethylation450BeadChip were used to interrogate genome-wide expression and methylation in tumor samples. Differential expression and methylation were analyzed through comparing tumors with adjacent non-tumor tissues. LncRNAs expressed differentially and correlated with coding genes and DNA methylation were validated in additional tumor samples using RT-qPCR and pyrosequencing. *In vitro* experiments were performed to evaluate lncRNA’s effects on tumor cells. We identified 8,500 lncRNAs expressed differentially between tumor and non-tumor tissues, of which 1,504 were correlated with mRNA expression. Two of the lncRNAs, *LOC146880* and *ENST00000439577*, were positively correlated with expression of two cancer-related genes, *KPNA2* and *RCC2*, respectively. High expression of *LOC146880* and *ENST00000439577* were also associated with poor survival. Analysis of lncRNA expression in relation to DNA methylation showed that *LOC146880* expression was down-regulated by DNA methylation in its promoter. Lowering the expression of *LOC146880* or *ENST00000439577* in tumor cells could inhibit cell proliferation, invasion and migration. Analysis of microarray data on gene expression and methylation allows us to identify two lncRNAs, *LOC146880* and *ENST00000439577,* which may promote the progression of NSCLC.

Lung cancer is one of the most common malignant tumors worldwild. According to the GLOBOCAN 2012, around 1.8 million people are diagnosed with lung cancer every year, consisting of 13% of all newly diagnosed cancer patients. Lung cancer is the number one cause of cancer deaths, about 1.6 million deaths a year, accounting for 19% of all cancer deaths^1^. Non-small cell lung cancer (NSCLC) is the most common form of lung cancer, which accounts for approximately 85% of all lung cancer cases. Because of the high mortality, NSCLC is one of the mostly studied malignancies. Despite extensive research, our understanding of the disease remains limited. One of the challenges is tumor heterogeneity due not only to individual’s variations in disease characteristics, but also cellular differences in response to therapeutic regimens. Rapid advancement in high-throughput technologies has provided new tools to study the disease in a more comprehensive fashion. Many researchers have begun to integrate omics data from different high-throughput platforms to investigate the disease mechanisms and to search for robust biomarkers^2^,^3^,^4^,^5^,^6^.

Epigenetic regulation of coding and non-coding genes has been shown to play important roles in genome functions. Research has revealed significant interactions betweeen and alterations in DNA methylation and gene expression in many types of cancer, including the breast^7^, prostate^8^, and lung^9^. Besides DNA methylation, non-coding RNA transcripts are also essential components of epigenetic regulation. Recently, long non-coding RNAs (lncRNAs) have drawn much attention in cancer research as this class of transcripts has complex and diverse biologic actions on cell activities and functions^10^. LncRNAs regulate gene expression through various mechanisms, including complementary binding to DNA, as well as to other coding and non-coding RNA transcripts^11^. Although there are significant interactions between DNA methylation and expression of protein-coding and non-coding RNAs, little is known about their alterations in lung cancer. An integrated genome-wide analysis of both DNA methylation and expression of lncRNAs and mRNAs may help to understand the complex regulatory network that underlies the tumorigenic mechanisms of the lung.

We report here our study of integrated genome-wide analyses of DNA methylation, lncRNA and gene expression in search for new regulatory and functional connections among DNA methylation, lncRNA and mRNA expression in lung cancer. Through exploring novel networks and mechanisms, we anticipate to identify new biomarkers and targets for lung cancer diagnosis, prognosis and treatment. Our study also offers clues for integration of genome-wide data from different platforms which have cross-talk in biological signaling and function, and advocates the use of this approach to further investigation of tumorigenesis and search for reliable tumor markers for clinical management of cancer patients.

## Results

### Identification of lncRNA-mRNA pairs

**Figure 1:**
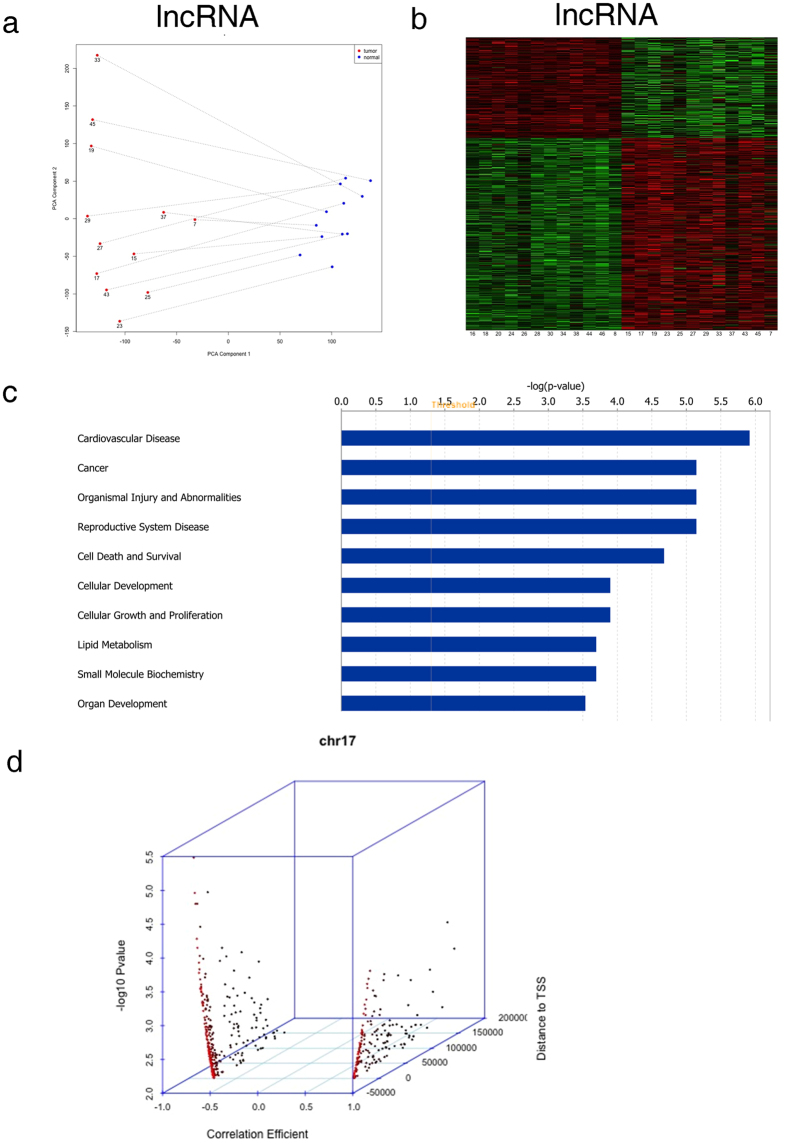
Bioinformatics analysis of lncRNA. (**a**) PCA of all lncRNA expression results from the microarray analysis. (**b**) Heatmap results based on the top 3,690 lncRNAs significantly associated with tumor samples from the microarray analysis of gene expression. (**c**) IPA results based on the 1,345 cis-pairs of lncRNAs and mRNAs which were differentially expressed in NSCLC. (**d**) Distributions of the methylation loci that were significantly correlated with lncRNA expression and their associated lncRNAs on chromosome 17. X axis is the correlation coefficient; Y axis is the p value; Z axis is the location of CpG sites in reference to TSS.

The workflow of our microarray analysis, data integration, gene identification and bioinformatic evaluation is shown in Supplementary Fig. S1. From the expression microarray, we found 10,206 transcripts differentially expressed between tumor and adjacent non-tumor tissues (*P *< 0.05 after FDR adjustment). Of these transcripts, 8,500 were lncRNAs and 1,685 were mRNAs. PCA plots (Fig. 1a) show complete separations of tumor and non-tumor samples in terms of lncRNA expression profiles. From the profiles, we selected top 3,690 lncRNAs to show the differences in lncRNA expression between tumor and non-tumor tissues, and the expression patterns were clearly different between tumor and non-tumor samples (Fig. 1b).

We next investigated the correlation of differentially expressed lncRNAs with differentially expressed mRNAs in NSCLC. The analyses yielded 1,504 pairs of lncRNAs and mRNAs with R^2^ > 0.5 (*P *< 0.05). Of these pairs, 1,345 were in cis- and 159 were in trans-correlation. IPA was performed on the cis-pairs. The top ten significant pathways were shown in Fig. 1c, and nearly half of the pathways were directly related to cancer, including cell death and survival, cellular development, and cellular growth and proliferation, suggesting the critical involvement of lncRNAs in tumorigenesis. From the list of correlated lncRNAs and mRNAs which also had large fold-change in expression and known biological annotation, we selected two pairs of lncRNAs and mRNAs (*LOC146880/KPNA2* and *ENST00000439577/RCC2*) for further validation. The coding genes in these pairs are known to have significant biological implications in NSCLC. The chromosomal location of the lncRNAs and their correlated genes are shown in Supplementary Fig. S2.

### Identification of lncRNA-methylation pairs

In the genome-wide methylation analysis, we identified 113,644 CpG with differential methylation (DM) between tumor and adjacent non-tumor tissues (*P *< 0.05 after FDR adjustment). We performed correlation analysis for the differentially expressed lncRNAs with their DM loci corresponding to the same chromosome. Among these DM loci, one methylation site (cg12562461) was located in the promoter of LOC146880 on chromosome 17. Distribution of the methylation sites and their associated lncRNAs on chromosome 17 revealed that more methylation sites around TSS were correlated with lncRNA expression (Fig. 1d). Results suggested that cg12562461 methylation was significantly lower in tumor than in non-tumor tissues, and the expression of *LOC146880* was inversely correlated with methylation.

### Validation of lncRNA/mRNA relations and analysis of their associations with survival

**Figure 2:**
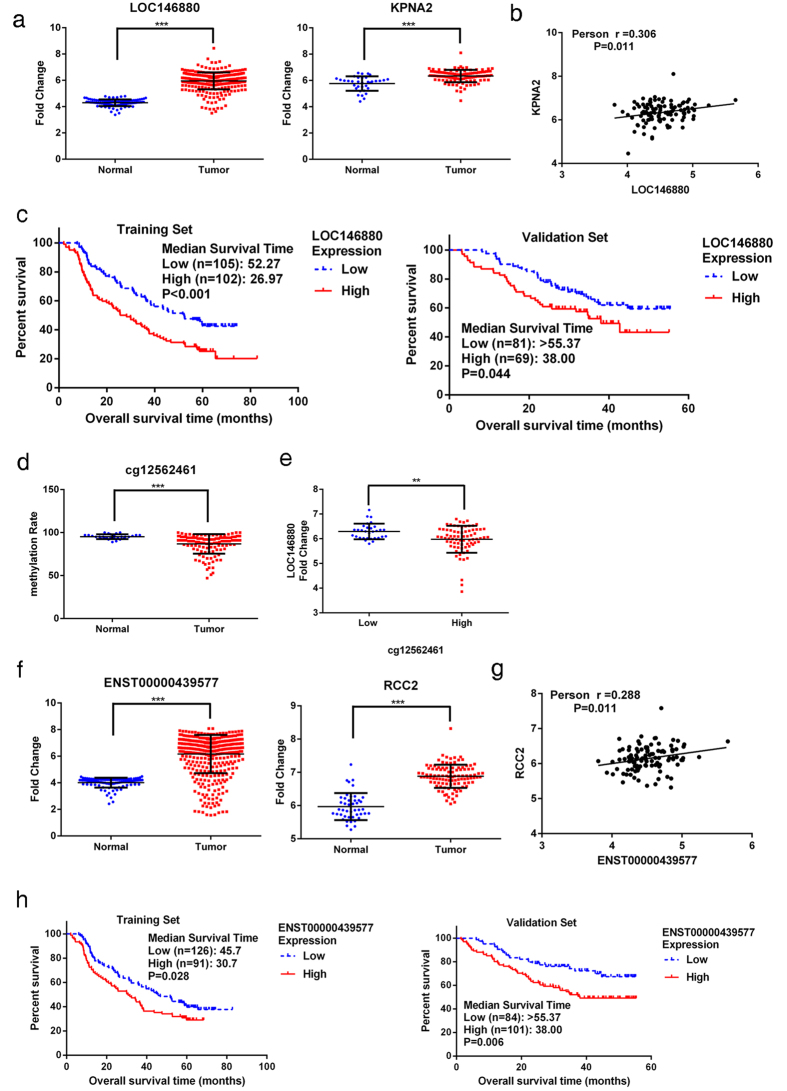
Validation of *LOC146880* and *ENST00000439577* expression in tumor and adjacent non-tumor tissues of NSCLC. (**a**) Higher expression of *LOC146880* and *KPNA2* in tumor than in adjacent non-tumor tissues. (**b**) A positive correlation between *LOC146880* and *KPNA2* expression. (**c**) High *LOC146880* expression associated with poor overall survival (left: training set; right: validation set). (**d**) Lower methylation of cg12562461 in tumor than in adjacent non-tumor tissues. (**e**) Lower methylation of cg12562461 in high than in low *LOC146880* expression tumors. (**f**) Higher expression of *ENST00000439577* and *RCC2* in tumor than in adjacent non-tumor tissues. (**g**) A positive correlation between *ENST00000439577* and *RCC2* expression. (**h**) High *ENST00000439577* expression associated with poor overall survival (left: training set; right: validation set). Data are expressed as mean ± s.d.; *P < 0.05; **P < 0.01; ***P < 0.001.

Using qPCR, we validated our microarray findings of two pairs of correlated lncRNAs and mRNAs in total 402 tumors and 105 adjacent non-tumor tissues. The qPCR results shown in Fig. 2 were similar to those of microarray results, showing that *LOC146880* and *KPNA2* expression was higher in tumor than in adjacent non-tumor tissues (Fig. 2a). We also observed a significant correlation between *LOC146880* and *KPNA2* expression (r = 0.306, *P *= 0.011) (Fig. 2b). We further analyzed the associations of *LOC146880* and *ENST00000439577* expression with patient’s clinical and pathological characteristics, and found that *LOC146880* expression was associated with histological type and tumor size, while *ENST00000439577* expression was associated with metastasis status and disease stage (Table 1). Kaplan-Meier survival analysis revealed that high expression of *LOC146880* was associated with poor overall survival both in the training (*P *< 0.001) and validation sets (*P *= 0.044) (Fig. 2c). In addition, we analyzed the methylation of cg12562461 located in TSS200 of *LOC146880* in 126 NSCLC tumors and 30 adjacent non-tumor tissues, and found low cg12562461 methylation in tumor than in adjacent non-tumor tissues (Fig. 2d). Our analysis also showed low *LOC146880* expression in high methylation and high expression in low methylation tissue samples (*P  *< 0.01) (Fig. 2e). Similarly, *ENST00000439577* and *RCC2* expression were higher in tumor than in adjacent non-tumor tissues (Fig. 2f), and *ENST00000439577* and *RCC2* expression were positively correlated (r = 0.288, *P *= 0.011) (Fig. 2g). Furthermore, high expression of *ENST00000439577* was associated with poor overall survival both in the training (*P *= 0.028) and validation sets (*P *= 0.006) (Fig. 2h).

### Functional analysis of *LOC146880* in NSCLC cell lines

**Figure 3:**
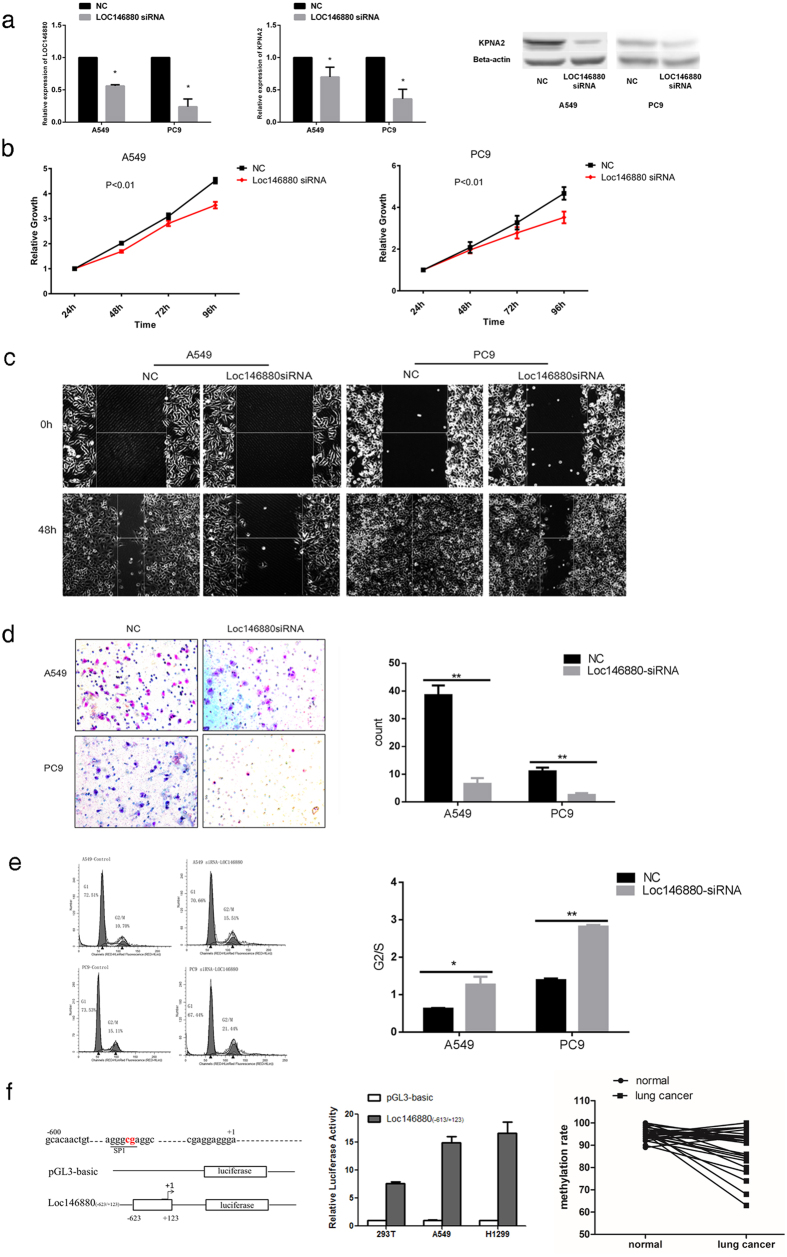
Functional analysis of *LOC146880* expression in NSCLC cell lines. (**a**) Declined expression of *LOC146880* and *KPNA2* after siRNA-*LOC146880* transfection. Declined expression of KPNA2 proteins after siRNA-*LOC146880* transfection. (**b**) Declined A549/PC9 cell proliferation after lowering the expression of *LOC146880*. (**c**) Declined A549/PC9 cell migration after lowering the expression of *LOC146880*. (**d**) Declined A549/PC9 cell invasion after lowering the expression of *LOC147880*. (**e**) Reduced cell cycle progression measured by the C6 flow cytometry after lowering the expression of *LOC146880*. (**f**) A promoter region of *LOC146880* contains a CpG site which was predicted to be a transcription factor SP1 binding site. A *LOC146880* fragment (−623/+123) was cloned into the pGL3-basic vector. The vector containing the *LOC146880* (−623/+123) construct displayed 15-fold increases in promoter activities compared with the PGL3-basic vector. *LOC146880* methylation is lower in tumors than in normal tissues. Data are expressed as mean ± s.d.; *P < 0.05, **P < 0.01. NC, Negative Control.

To examine the biological relevance of *LOC146880* in lung cancer, we analyzed baseline expression of *LOC146880* in three NSCLC (A549, PC9 and H1299) and one normal cell lines (Beas2B). Our analysis showed that *LOC146880* expression was high in A549 and PC9 compared to Beas2B, but low in H1299 (see Supplementary Fig. S3). Based on these results, we selected A549 and PC9 for our knockdown experiments. We made a small interfering RNA against *LOC146880*, siRNA-*LOC146880*, and transfected the siRNA into A549 and PC9 cell lines. Transfected cells showed significant decreases in *LOC146880* expression (Fig. 3a). As a downstream molecule, KPNA2 expression were also declined in A549 and PC9 at both mRNA and protein levels (Fig. 3a). Moreover, low expression of *LOC146880* inhibited both A549 and PC9 proliferation (Fig. 3b). Furthermore, knockdown of *LOC146880* by siRNA significantly inhibited cell migration (Fig. 3c) and invasion (Fig. 3d) in A549 and PC9. Flow cytometry analysis showed that lowering *LOC146880* expression slowed down cell cycle progression at the G2/M phase (Fig. 3e), suggesting that declined *LOC146880* expression may put a break on cell cycle progression resulting in suppression of cell proliferation and migration. Taken together, our findings indicate that *LOC146880* may function as an oncogenic transcript in NSCLC.

Based on the prediction by TRANSFAC^®^ (http://www.gene-regulation.com/pub/databases.html), *LOC146880* promoter contains one CpG site which is located in the transcription factor (SP1) binding site, suggesting that methylation in the region may block the SP1 binding to the promoter, resulting in suppression of *LOC146880* expression. To test this possibility, We inserted a *LOC146880* promoter construct (−623/+123) into a plasmid (pGL3), and transfected it into HEK293T, A549 and H1299 cell lines which were co-transfacted with a SP1 overexpression vector. Compared to the control (pGL3-basic), *LOC146880* transfected cells (pGL3-*LOC146880*) displayed significant promoter activities, suggesting the existence of SP1 activation in the *LOC146880* promoter (Fig. 3f).

**Figure 4:**
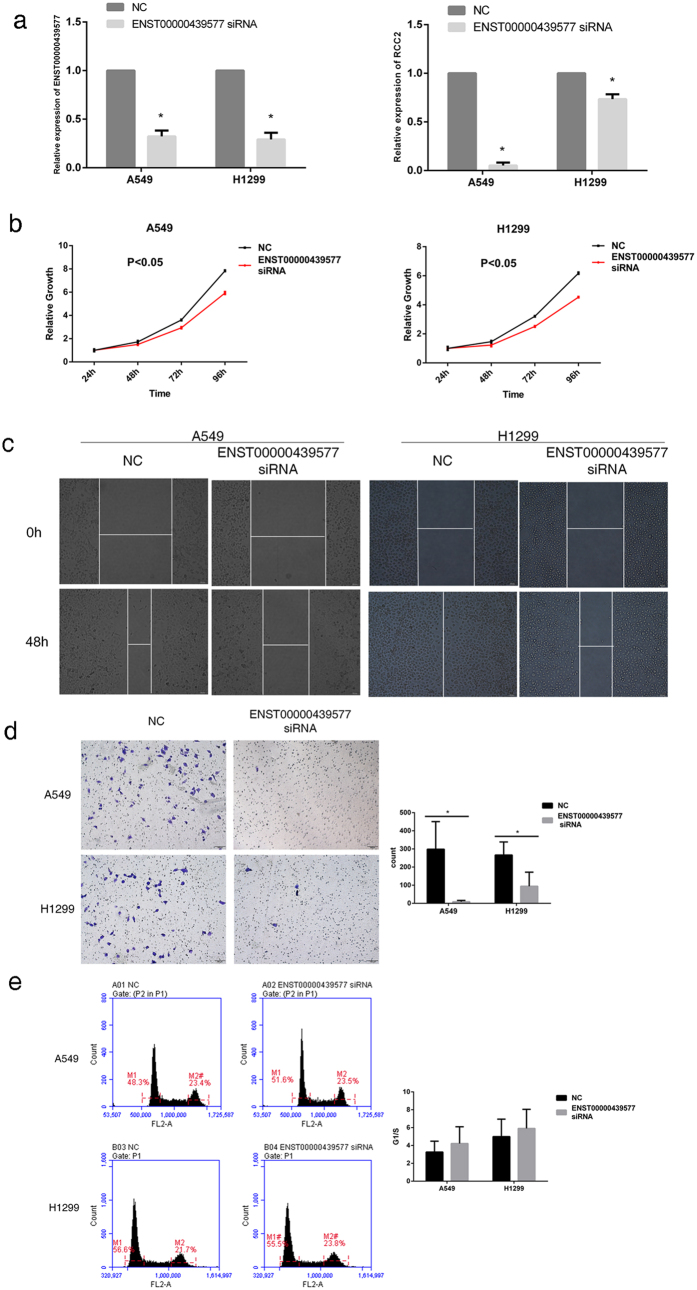
Functional analysis of *ENST00000439577* expression in NSCLC cell lines A549 and H1299. (**a**) Declined expression of *ENST00000439577* and *RCC2* after siRNA-*ENST00000439577* transfection. (**b**) Reduced A549/H1299 cell proliferation after lowering the expression of *ENST00000439577*. (**c**) Reduced A549/H1299 cell migration after lowering the expression of *ENST00000439577*. (**d**) Reduced A549/H1299 cell invasion after lowering the expression of *ENST00000439577*. (**e**) Cell cycle analysis by the C6 flow cytometry after lowering the expression of *ENST00000439577*. Data are expressed as mean ± s.d.; *P < 0.05. NC, Negative Control.

Baseline expression of *ENST00000439577* was measured in three NSCLC (A549, H1975 and H1299) and one normal cell lines (Beas2B). The analysis showed that *ENST00000439577* expression was high in A549, H1299 and H1975 compared to Beas2B (see Supplementary Fig. S4). A549 and H1299 cell lines were selected for testing the effects of *ENST00000439577*. We made a small interfering RNA against *ENST00000439577*, siRNA-*ENST00000439577*, and transfected the siRNA into A549 and H1299 cell lines. After transfection, cells showed significant declines in *ENST00000439577* expression. *RCC2* expression was also reduced in A549 and H1299 (Fig. 4a). Low expression of *ENST00000439577* inhibited both A549 and H1299 proliferation (Fig. 4b). Furthermore, knockdown of *ENST00000439577* by siRNA significantly inhibited cell migration (Fig. 4c) and invasion (Fig. 4d) in A549 and H1299. Flow cytometry analysis indicated that cell cycle pregresion was slightly reduced at the G2/M phase in both cell lines after lowering *ENST00000439577* expression, although the changes were not statistically signigicant (Fig. 4e). We also analyzed CpG site methylation in the promoter of ENST00000439577, and found no significant correlation between methylation and expression.

## Discussion

With the rapid improvement in our understanding of the human genome from nucleotide sequences to functional regulation, we now recognize that changes in epigenetic regulation, including histone modifications, DNA methylation and non-coding RNA expression, play key roles in the development and progression of human cancer. High-throughput technology allows us to analyze genome-wide changes of DNA methylation and gene expression in cancer, but the challenge is how to effectively use the massive high-dimensional data to understand the process of tumorigenesis.

Although much research on DNA methylation and gene expression has been directed towards the understanding of protein-coding genes and their functions, evidence also suggests that we should integrate multiple layers of epigenetic regulation to reveal the well-orchestrated regulatory network that is beyond the production and activities of proteins. In this study, we performed genome-wide analyses of lncRNA, mRNA expression and DNA methylation, and integrated the two platforms of high-dimensional data to evaluate concurrent changes between epigenetic regulation and gene expression in NSCLC. Integrating the expression profiles of both coding and non-coding RNAs with genome-wide DNA methylation may also aid our understanding of the interactions between epigenetic modifications and gene expression in lung cancer. Furthermore, this integrated approach may help to discover and develop novel epigenetic biomarkers for diagnosis, prognosis and treatment of lung cancer.

In our study, we discovered two novel lncRNAs whose expression were significantly elevated in NSCLC, and high expression were associated with poor overall survival as well as with two possible onco-proteins. High expression of *LOC146880* was associated with high expression of *KPNA2*, and suppressing *LOC146880* expression resulted in declines in *KPNA2* expression in NSCLC cell lines. *KPNA2* belonges to the karyopherin α family, which has been reported as a key nucleocytoplasmic transport protein in the translocation of several cancer-related proteins. *KPNA2* may play critical roles in carcinogenesis, cell differentiation, transcription regulation and immune response. Recently studies have demonstracted that high expression of *KPNA2* is associated with poor prognosis of esophageal squamous cell carcinoma^12^, epithelial ovarian carcinamos^13^, gastric cancer^14^, prostate cancer^15^, upper tract urothelial carcinoma^16^ and breast cancer^17^. Teng *et al*. found that KPNA2 was involved in DNA damage repair through mediating the NBS1 subcellular location and functions of the MRE11-RAD50-NBS1 complex in tumorigenesis^18^. It was also suggested that silencing *KPNA2* could decrease the nuclear translocation of POU class 5 homeobox 1 (Oct4), suppressing the proliferation of lung cancer cells^19^. We speculate that *LOC146880* may play a critical role in tumorigenesis through the regulation of *KPNA2* expression. In our analysis, we found not only *LOC146880* expression in NSCLC being associated with patient survival outcomes, but also changes of its expression in lung tumor cells resulting in downregulation of *KPNA2* as well as suppression of cell proliferation, migration and invasion. In our investigation, we also found that the transcription activity of *LOC146880* was affected by promoter methylation.

*ENST00000439577* and *RCC2* were another pair of correlated lncRNA/mRNA expression associated with NSCLC. High *ENST00000439577* expression was associated with high expression of *RCC2*, as well as with poor overall survival of NSCLC patients. We knocked down the expression of *ENST00000439577* using siRNA, and found decreases in the expression of *RCC2* in NSCLC cell lines, which resulted in inhibition of cell proliferation, migration and invasion. Yenjerla *et al*. reported that TD-60 (also known as RCC2) was a mitotic centromere-associated protein and an essetial regulator of cell cycle progression through G_1_, S, and G_2_ phases into mitosis^20^,^21^. Moreover, TD-60 displayed high guanine exchange factor (GEF) activity for the Ras-related protein RalA, and regulated the kinetochore-microtubule stability in early mitosis^22^. Subsequent studies revealed that Ral GEFs palyed an important role during the Ras-induced tumourigenesis via activating the Ral GTPases^23^. Furthermore, down-regulation of RalA could reactivate p53 protein expression and stability, and up-regulate the expression of p53-downstream molecule *p21* in NSCLC cell lines H460 that harbored mutant K-*Ras* and wild-type *p53*^24^. Guin *et al*. found that RalA/RalB, as the downstream effector of RAS, might be important for tumor metastasis and invasion of NSCLC, but, however, their mechanisms remained unknown^25^. According to our results, we speculate that high expression of *ENST00000439577* could up-regulate *RCC2* expression, which may promote the activity of RalA, stimulating the growth and invasion of lung tumour cells. Consequently, lncRNA *ENST00000439577*, which influences the Ral signalling pathways via *RCC2*, may be a potential therapeutic target for the treatment of lung cancer.

Although we used a limited discovery set to identify lncRNAs and methylation loci, we were able to validate our microarray results in additional specimens. This process demonstrates that microarray is quite efficient in identifying potential molecules involved in lung cancer. In addition to the integrated analysis of lncRNAs and mRNAs expression, we also investigated the correlation of lncRNAs expression and their methylation in lung cancer. All the analyses indicate that our approach to integrate different microarray analyses is clinically and biologically relevant and that our findings of expression of coding and non-coding RNAs and methylation loci may help to develop useful biomarkers for prognosis and treatment of lung cancer. More studies are needed to further explore and integrate different high-dimensional data. These approaches will help to generate valuable insights into the molecular mechanisms of lung tumorigenesis and progression.

## Methods

### Study patients

NSCLC patients (n = 490) were recruited from the Tianjin Medical University Cancer Hospital (TMUCH) between May 2006 and July 2011. These patients were divided into two groups: one training set including patients recruited before December 31 2008, one validation set including patients recruited after January 1, 2009. Informed consents were obtained from all the subjects. Fresh tumor samples and adjacent non-tumor tissues were collected from the patients during their surgery. Twelve pairs of tumor and adjacent non-tumor samples were used for microarray analyses of DNA methylation and gene expression, and 217 tumor samples from the training set and 185 tumor samples from the validation set were analyzed for expression of lncRNAs using qPCR, respectively, and 126 tumor samples were analyzed for validation of methylation using pyrosequencing. Patients’ characteristics are shown in Supplementary files (see Supplementary Table S1). All the patients were followed from surgery (earliest in May 2006) to August 2013. The study was approved both by the Medical Ethical Review Committees at TMUCH and Shanghai Jiao Tong University School of Public Health. These experimental protocols were carried out in accordance to with the approved guidelines of TMUCH and Shanghai Jiao Tong University School of Public Health.

### Microarray of gene expression

Expression of lncRNAs and mRNAs were measured using a SBC microarray chip (Shanghai Biotechnology Company, Shanghai, China), which was based on the Agilent eArray platform with additional probes to lncRNAs. The chip detects 48,011 lncRNA and 41,712 mRNA transcripts. Data were extracted using the Feature Extraction software 10.7 (Agilent technologies, Santa Clara, CA). Raw data were normalized with the Quantile algorithm, Gene Spring Software 11.0 (Agilent technologies, Santa Clara, CA).

### Microarray of DNA methylation

DNA methylation was measured in 12 paires of tissue samples using the Illumina HumanMethylation450 BeadChip (Illumina, San Diego, CA). The chip analysis of DNA methylation has been described elsewhere^26^.

### DNA and RNA extraction

Genomic DNA and total RNAs were isolated from tissue samples using the QIAamp DNA Mini Kit (Qiagen, Hilden, Germany) and TRIzol reagents (Invitrogen, Grand Island, NY), respectively, according to the manufacturer’s protocols. DNA and RNA concentrations were quantified with UV light using a Nanodrop ND-8000 spectrophotometer (Nanodrop Technologies, Wilmington, DE).

### Reverse-transcriptase quantitative PCR (RT-qPCR)

Total RNA was reversely transcribed to cDNA using the PrimerScript RT Kit with the gDNA Eraser (Takara, Dalian, China) in accordance with the manufacturer’s instructions. Beta-actin was used as an internal control. For data analysis, the qPCR results were quantified as fold changes between Beta-actin and target genes using the formula 2^−△△Ct^.

### DNA extraction and pyrosequencing analysis of DNA methylation

Bisulfite pyrosequencing was performed to validate our microarray results in tumor samples. Genomic DNA was bisulfite-converted using the EpiTect plus DNA Bisulfite Kit (Qiagen, Hilden, Germany) according to the manufacturer’s protocol. The treated DNA samples were PCR-amplified and fragmented. The processed samples were then precipitated, suspended and sequenced in the Pyro Mark Q96 system (Qiagen, Hilden, Germany). Primer sequences and PCR conditions are provided in Supplementary files (see Supplementary Table S2). The primers were designed to include CpG sites within 0.5 kb of the transcription start site. A methylation level >15% was considered positive for methylation. Any levels equal to or lower than that could not be easily distinguished from the background, and therefore were regarded as negative for methylation.

### Cell culture

Normal cell line HEK293T, Beas2B and NSCLC cell lines A549, H1299, H1975 and PC9 were purchased from the Shanghai Cell Bank, Type Culture Collection Committee of Chinese Academy of Science (CAS, Shanghai, China) and were authenticated using short tandem repeat profiling by the cell bank in 2015. These cells were maintained in RPMI-1640 medium supplement with 10% fetal calf serum (Gibco, Grand Island, NY) at 37 °C in a humid atmosphere containing 5% CO_2_ according to the recommendation by CAS. We only used cells within 6 months from being thawed for the current study and recorded the cell morphology and doubling times regularly to ensure the maintenance of phenotypes.

### Cell transfection experiments

To knockdown the expression of *LOC146880* or *ENST00000439577*, siRNA-*LOC146880* or siRNA-*ENST00000439577* were transfected into the NSCLC cell lines using the Lipofectamine^®^ 2000 Reagent (Invitrogen, Grand Island, NY) following the manufacturer’s instructions. We designed three specific siRNAs for knockdown of each target of interest and mixed them for transfection. The siRNAs information is shown in Supplementary Table S3. For comparison, siRNA-negative control was also transfected under the same condition.

### Luciferase report assay

To determine if *LOC146880* expression was regulated by promoter methylation, a 746 bp PCR product containing the *LOC146880* promoter (from −623 to +123 relative to the transcription start site) was cloned into the pGL3 luciferase report vector (Promega, Madison, WI) between the restriction sits KpnI and HindIII. HEK293T and NSCLC cell lines (H1299 and A549) were seeded onto 24-well plates at 5 × 10^4^ cells per well for 24 hours prior to transfection. The cells were transfected either with 0.5 mg pGL3-*LOC146880* (−623/+123) or pGL3-basic (control) using the Lipofectamine^®^ 2000 Reagent according to the manufacturer’s instructions. PRL-TK plasmid (10 ng) containing the *Renilla* luciferase gene (Promega, Madison, WI) was co-transfected to standardize transfection efficiency. The relative luminescence was measured 24 hours post-transfection using the Dual-Luciferase Reporter Assay System (Promega, Madison, WI). The experiments were performed in triplicate.

### Assays of cell proliferation, wound healing, trans-well mobility and cell cycle progression

These assays have been described elsewhere^27^.

### Western blot assay

Cultured cells were lysed on ice for 30 minutes in 1x RIPA Lysis Buffer supplemented with a protease inhibitor cocktail (Millipore, Billerica, MA). Protein concentrations of the cell lysates were measured with the BCA Protein Assay Kit (Sangon Biotech, Shanghai, China). Denatured proteins (40 μg) were electrophoresed on 10% SDS-PAGE gel and were electrotransferred from the gel to PDVF membranes (Millipore, Billerica, MA). After blocking with 5% non-fat milk in a Tris buffered saline with 0.05% Tween-20 for one hour at room temperature, the membranes were incubated overnight at 4 °C with primary antibodies against KPNA2 (Abcam, Cambridge, MA) at a dilution of 1:500, and β-actin 1:1000 (Santa Cruz Biotechnology, Dallas, TX). Secondary antibodies were added at concentrations of 1:4000. ECL Kit (Millipore, Billerica, MA) was used to visualize protein expression.

### Microarray data analysis

Expression data were evaluated for reliability with reference to internal controls. Raw data were normalized with Quantile algorithm, Gene Spring 11.0 (Agilent technologies, Santa Clara, CA). Limma package in R (3.2.2 version) was used to determine differential expression of lncRNA and mRNA between matched tumor and non-tumor samples, followed by the Benjamin-Hochberg FDR correction. Correlations between lncRNAs and mRNAs were analyzed using R (3.2.2 version). LncRNAs were annotated for their protein-coding targets based on 1) their proximities to the protein coding genes (cis-targets) and 2) sequence homologies determined by BLAST (trans-targets). Heatmap was generated to show expression patterns. Principal component analysis (PCA) was performed to assess sample clustering. Analysis of functional enrichment on selected genes was performed using the Ingenuity Pathway Analysis (IPA) (http://www.ingenuity.com; September 2015).

Correlations between lncRNA expression and DNA methylation were analyzed for the methylation sites located within the lncRNA genes or corresponding to the TSS1500 regions. To show the patterns of these relationships, correlation coefficients with *p* values less than or equal to 0.01 were selected for plot. For each chromosome, the correlation’s *P* values, correlation coefficients and distances to TSS were plotted.

### Statistical analysis

SPSS software version 16.0 was used for statistical analysis. Student’s *t*-test was performed to compare changes in expression. Log-rank test and Kaplan-Meier survival plot were used to evaluate associations of overall survival with methylation or expression of lncRNAs or coding genes. Cox proportional hazards regression was employed for survival analysis with adjustment for covariates. All statistical tests were two-tailed. Overall survival was calculated from the date of surgery to the date of death or last live contact.

## Additional Information

**How to cite this article**: Feng, N. *et al*. Analysis of Microarray Data on Gene Expression and Methylation to Identify Long Non-coding RNAs in Non-small Cell Lung Cancer. *Sci. Rep.* **6**, 37233; doi: 10.1038/srep37233 (2016).

**Publisher’s note:** Springer Nature remains neutral with regard to jurisdictional claims in published maps and institutional affiliations.

## Supplementary Material

Supplementary Information

## Figures and Tables

**Table 1:** Associations of *LOC146880* and *ENST00000439577* expression with patient’s clinical and pathological characteristics.

Characteristics		No.	*LOC146880* mean + s.d.	*P*	No.	*ENST00000439577* mean + s.d.	*P*
No. of Sample		357			402		
Age at diagnosis (years)	≤60	170	6.052 + 0.632	0.759	186	6.204 + 1.410	0.574
	>60	187	6.033 + 0.560		216	6.123 + 1.466	
Gender	Male	208	6.085 + 0.459	0.134	240	6.109 + 1.499	0.386
	female	149	5.982 + 0.741		162	6.236 + 1.345	
Lung disease history	No	332	6.039 + 0.598	0.695	374	6.152 + 1.436	0.695
	Yes	25	6.087 + 0.546		28	6.263 + 1.491	
family history	No	290	6.041 + 0.599	0.938	327	6.207 + 1.434	0.137
	Yes	65	6.048 + 0.581		72	5.928 + 1.468	
Smoking status	No	147	5.965 + 0.754	0.060	157	6.205 + 1.373	0.617
	Yes	210	6.096 + 0.444		245	6.131 + 1.481	
Histological type	SCC	139	6.099 + 0.415	0.054	165	6.168 + 1.405	0.497
	ADC	162	5.937 + 0.708	**0.017**^a^	177	6.051 + 1.512	0.460^a^
	SCC&ADC	26	6.044 + 0.433		26	5.832 + 1.525	
Tumor size	T1	131	5.933 + 0.694	**0.014**	145	6.083 + 1.452	0.432
	T2/3/4	223	6.106 + 0.520		254	6.201 + 1.438	
Lymph node status	N0	180	6.023 + 0.636	0.284	204	6.004 + 1.508	**0.027**
	N1/2/3	164	6.089 + 0.495		185	6.327 + 1.349	
Metastasis status	M0	334	6.041 + 0.572	0.865	378	6.131 + 1.462	**0.021**
	M1	23	6.063 + 0.874		24	6.613 + 0.899	
Disease stage	I/Π	203	6.004 + 0.615	0.139	239	6.012 + 1.506	**0.007**
	III/IV	146	6.099 + 0.563		155	6.400 + 1.286	

^a^All the statistical tests were based on the Student’s t test, except the histology which was based on ANOVA test. The difference between SCC and ADC was compared using the LSD test.
