# Supplementary material for: Analysis of Microarray Data on Gene Expression and Methylation to Identify Long Non-coding RNAs in Non-small Cell Lung Cancer
Source: Sci Rep. 2016 Nov 16;6:37233. doi: 10.1038/srep37233 (PMC5110979; doi:10.1038/srep37233)

**Analysis of Microarray Data on Gene Expression and Methylation to Identify Long Non-coding RNAs in Non-small Cell Lung Cancer**

Nannan Feng^a§^, Travers Ching^b§^, Yu Wang^c^, Ben Liu^c^, Hongyan Lin^a^, Oumin Shi^a^, Xiaohong Zhang^a^, Min Zheng^a^, Xin Zheng^a^, Ming Gao^c^, Zhijie Zheng^a^, Herbert Yu^b^, Lana Garmire^b*^, Biyun Qian^a*^

**Running title:** LncRNAs in Lung Cancer

**Affiliations:**

a. Hongqiao International Institute of Medicine, Shanghai Tongren Hospital and Faculty of Public Health, Shanghai Jiao Tong University School of Medicine, Shanghai 200025, China.

b. Cancer Epidemiology Program, University of Hawaii Cancer Center, 701 Ilalo Street, Honolulu, HI 96813, USA.

c. Key Laboratory of Cancer Prevention and Therapy, Tianjin Medical University Cancer Institute and Hospital, Tianjin, 300060, China.

^§^ These authors contributed equally to this work.

^*^These authors jointly directed this work.

**Corresponding author:**

Biyun Qian, MD, PhD. Professor.

Hongqiao International Institute of Medicine, Shanghai Tongren Hospital and Faculty of Public Health, Shanghai Jiao Tong University School of Medicine, 227 South Chongqing Road, Shanghai 200025, China. Fax: 86-21-63850472. Email: [qianbiyun@shsmu.edu.cn](mailto:qianbiyun@shsmu.edu.cn).

Lana Garmire, PhD. Assistant Professor.

Cancer Epidemiology Program, University of Hawaii Cancer Center, 701 Ilalo Street, Honolulu, HI 96813, USA. Email: LGarmire@cc.hawaii.edu.

**Supplementary Table S1.** Baseline characteristics of the study population in screening and validation sets

SCC: squamous cell cancer; ADC: Adenocarcinoma; MD: max diameter

^a^ contains samples both of training set and validation set.

| Parameter |  | microarray | qRT-PCR^a^ | Pyrosequencing |
| --- | --- | --- | --- | --- |
| Tissue source |  | Fresh-frozen | Fresh-frozen | Fresh-frozen |
| No. |  | 12 | 402 | 126 |
| Age at diagnosis (years) | ≤60 | 8(67%) | 186(46%) | 52(41%) |
|  | >60 | 4(33%) | 216(54%) | 74(59%) |
| Gender | Male | 9(75%) | 240(59%) | 71(56%) |
|  | female | 3(25%) | 162(41%) | 55(44%) |
| BMI |  | 23.34+3.07 | 30.99+3.08 | 24.37+3.38 |
| Lung disease history | No | 12(100%) | 374(93%) | 115(91%) |
|  | Yes | 0(0%) | 28(7%) | 11(9%) |
| family history | No | 9(75%) | 327(82%) | 104(83%) |
|  | Yes | 3(25%) | 72(18%) | 21(17%) |
| Smoking status | No | 4(33%) | 157(39%) | 45(36%) |
|  | Yes | 8(67%) | 245(61%) | 81(64%) |
| Histological type | SCC | 8(67%) | 165(41%) | 62(49%) |
|  | ADC | 4(33%) | 177(44%) | 52(41%) |
|  | SCC&ADC | 0(0%) | 26(6%) | 4(3%) |
|  | other | 0(0%) | 34(9%) | 8(6%) |
| Tumor size | T1 | 3(25%) | 145(36%) | 42(33%) |
|  | T2/3/4 | 9(75%) | 254(64%) | 84(67%) |
| Lymph node status | N0 | 4(33%) | 204(52%) | 68(55%) |
|  | N1/2/3 | 8(67%) | 185(48%) | 55(45%) |
| Metastases status | M0 | 6(50%) | 378(94%) | 120(95%) |
|  | M1 | 6(50%) | 24(6%) | 6(5%) |
| Disease stage | I/Π | 8(67%) | 239(61%) | 82(66%) |
|  | Ⅲ/Ⅳ | 4(33%) | 155(39%) | 43(34%) |
| status | censored | 5(42%) | 189(47%) | 63(50%) |
|  | death | 7(58%) | 213(53%) | 63(50%) |

**Supplementary Table S2.** Primer sequences and polymerase chain reaction (PCR) conditions in Pyrosequencing

| LncRNA | Methylation site | Primer | |  |
| --- | --- | --- | --- | --- |
| *LOC146880* | cg12562461 | Forward | GGGGTTTTTTGTTTGGGAATTAT |  |
|  |  | Reverse-Bio | ACAACCACCTCCATAAACACAACTATT |  |
|  |  | Sequence | GGAATTATTTGTTTTTTTGGATAAG |  |

PCR condition as followed: 95 ℃ for 5 minutes, then 94℃ for 30 seconds, 56℃ for 30 seconds, 72℃ for 30 seconds for 45-48 cycles, then 72℃ for 10 minutes, 4℃ soak.

**Supplementary Table S3**. Information of siRNAs sequences for transfection

| LncRNA-siRNA |  | Sense/Anti-sense |
| --- | --- | --- |
| *ENST00000439577* | siRNA-1 | 5’ –CAAUGUUGUUGUUUAUUUAAA -3’/  5’ –UAAAUAAACAACAACAUUGUC -3’ |
|  | siRNA-2 | 5’ –AGACAAUUACAGCACUAAACC -3’/  5’ –UUUAGUGCUGUAAUUGUCUUA -3’ |
|  | siRNA-3 | 5’ –ACGCUAAGCCUCUUUCAAAUU -3’/  5’ –UUUGAAAGAGGCUUAGCGUUU -3’ |
| *LOC146880* | siRNA-1 | 5’ –GAGGAACGCUUGACAAAGUTT -3’/  5’ –ACUUUGUCAAGCGUUCCUCTT -3’ |
|  | siRNA-2 | 5’ –CGACCUUAGAGCAGGUAAUTT -3’/  5’ –AUUACCUGCUCUAAGGUCGTT -3’ |
|  | siRNA-3 | 5’ –GAGGCUGAAAUCUUAGAAUTT -3’/  5’ –AUUCUAAGAUUUCAGCCUCTT -3’ |
| Negative Control | siRNA-NC | 5’ -UUCUCCGAACGUGUCACGUTT -3’/  5’ -ACGUGACACGUUCGGAGAATT -3 |

**Supplemental Figure Legends**

**Supplementary Figure S1**. Flowchart of microarray data analysis and result

**Supplementary Figure S2.** The schematic figures of the location of the lncRNAs and their associated genes

**Supplementary Figure S3**. Baseline expression of *LOC146880* in Beas2B and NSCLC cell lines

**Supplementary Figure S4.** Baseline expression of *ENST00000439577* in Beas2B and NSCLC cell lines


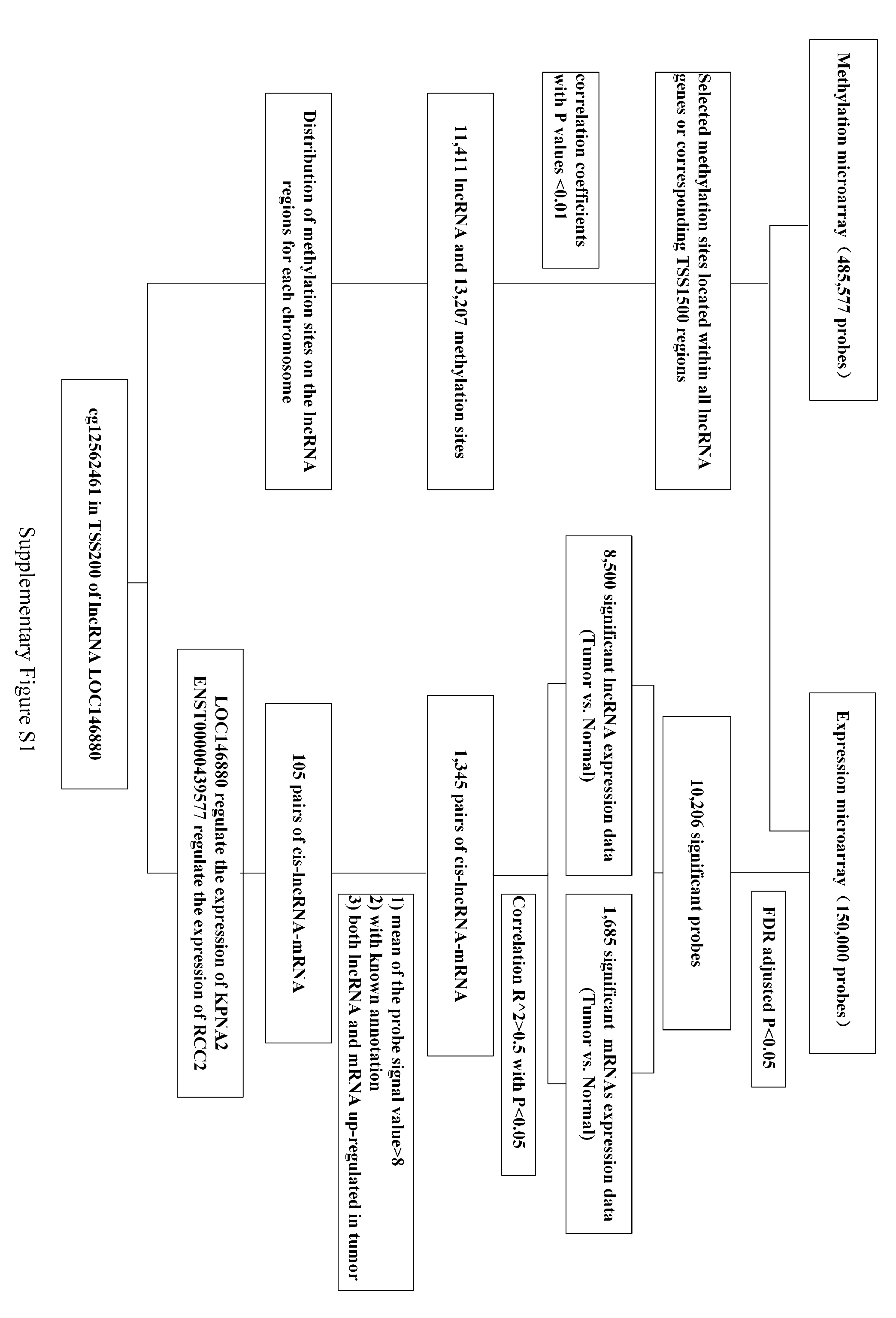


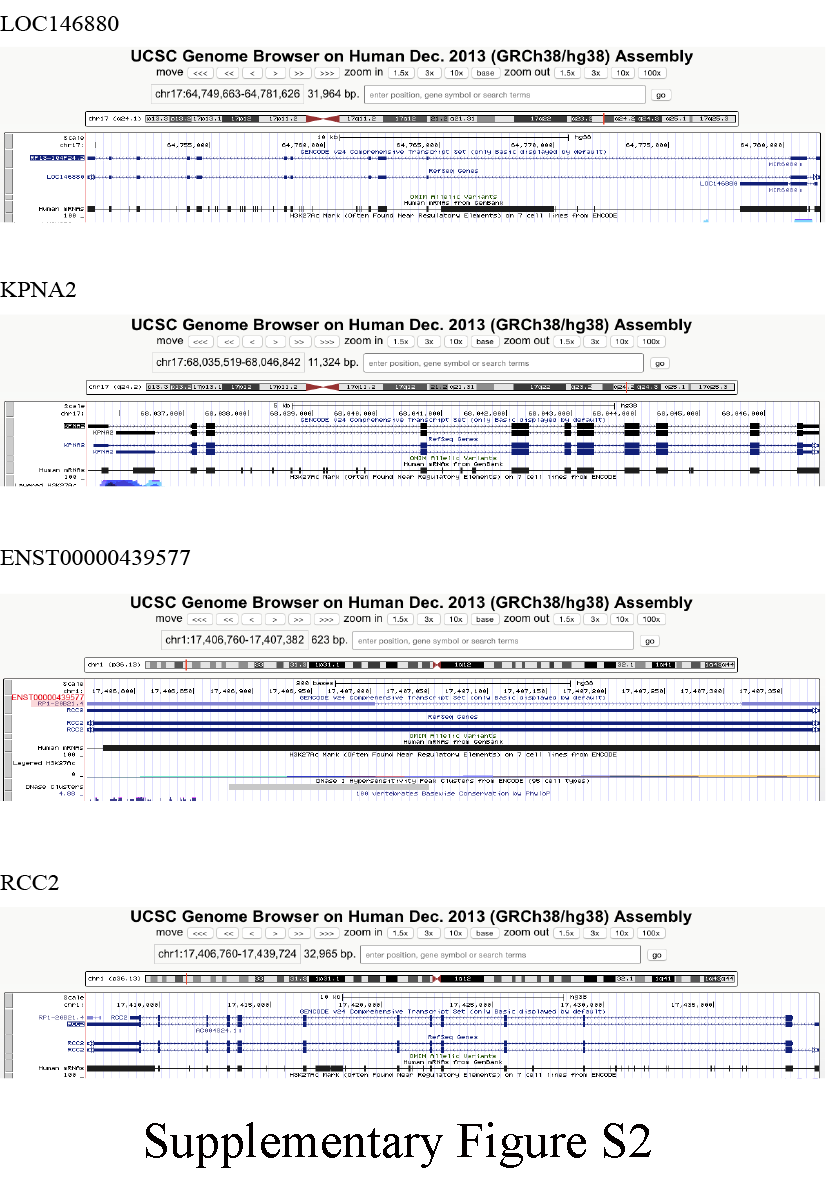


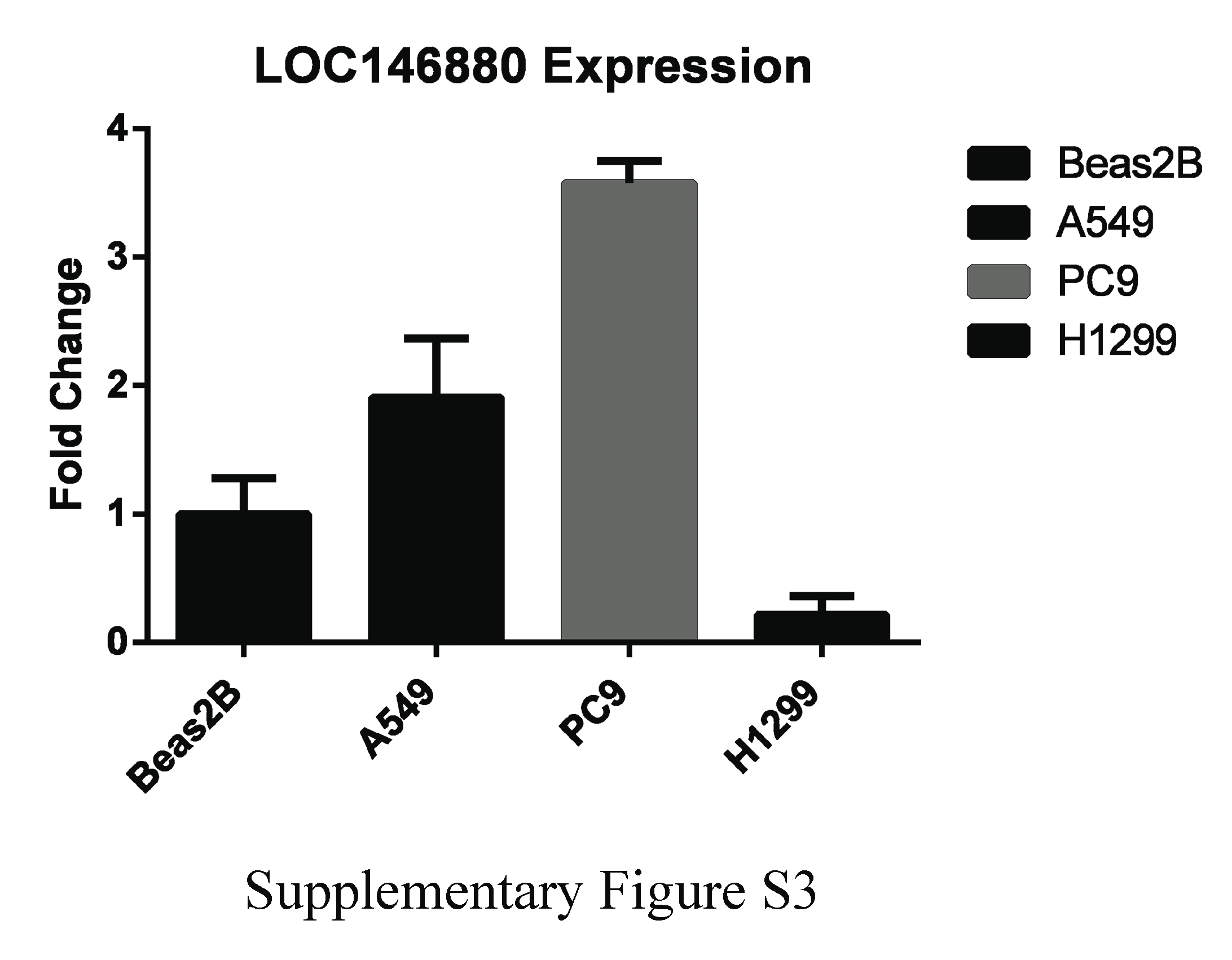


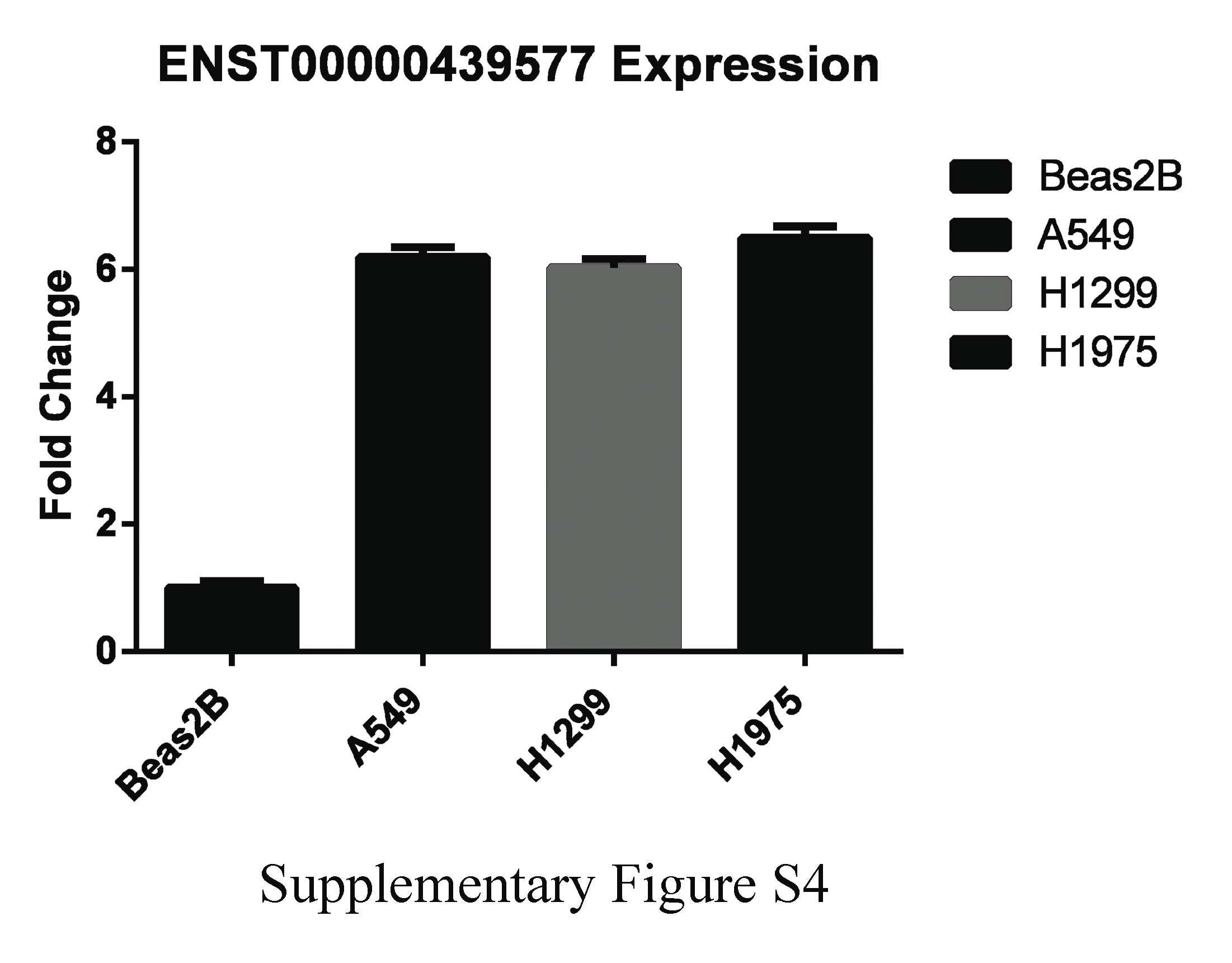

Supplement: Supplementary Information [file srep37233-s1.doc]
